# Efficacy of Rose Oil Soft Capsules on Clinical Outcomes in Ulcerative Colitis: A Pilot Randomized, Double-Blinded, Placebo-Controlled Clinical Trial

**DOI:** 10.31661/gmj.v8i0.1307

**Published:** 2019-07-07

**Authors:** Ali Tavakoli, Meysam Shirzad, Alireza Taghavi, Mohammadreza Fattahi, Mohammadmahdi Ahmadian-Attari, Leila Mohammad Taghizadeh, Mahsa Rostami Chaijan, Masih Sedigh Rahimabadi, Rahimeh Akrami, Mehdi Pasalar

**Affiliations:** ^1^Research Center for Traditional Medicine and History of Medicine, Shiraz University of Medical Sciences, Shiraz, Iran; ^2^Department of Traditional Medicine, Faculty of Medicine, Shiraz University of Medical Sciences, Shiraz, Iran; ^3^Department of Persian Medicine, School of Persian Medicine, Tehran University of Medical Sciences, Tehran, Iran; ^4^Gastroenterohepatology Research Center, Shiraz University of Medical Sciences, Shiraz, Iran; ^5^Evidence-based Phytotherapy and Complementary Medicine Research Center, Alborz University of Medical Sciences, Karaj, Iran; ^6^Department of Traditional Medicine, Medicinal Plants Research Center of Barij, Kashan, Iran; ^7^Department of Persian Medicine, Fasa University of Medical Sciences, Fasa, Iran; ^8^Essence of Parsiyan Wisdom Institute, Traditional Medicine and Medicinal Plant Incubator, Shiraz University of Medical Sciences, Shiraz, Iran

**Keywords:** Ulcerative Colitis, Rose Oil, Clinical Trial, Calprotectin

## Abstract

**Background:**

Ulcerative colitis is the most common form of inflammatory bowel disease worldwide, which presents with superficial ulcers in the rectum and colon. The aim of this study was to assess the effectiveness of rose oil soft capsules over placebo on the clinical outcomes in moderate to severe ulcerative colitis.

**Materials and Methods:**

This study was a pilot randomized, double-blind clinical trial, and the 40 patients were assigned into rose oil and placebo groups (n=20 per group). All patients were instructed to use their prescribed two soft capsules three times daily for two months. The clinical symptoms, quality of life the patients, and calprotectin level were evaluated via partial Mayo clinic score, irritable bowel disease questionnaire (IBDQ-9), and calprotectin kit as primary outcome measures.

**Results:**

The mean age of the participants was 41±10 years. Most of them (53.6%) were male, and the remaining (46.4%) were female. The demographic and baseline data showed no differences between the two groups. Partial Mayo clinic scores decreased in both groups after the treatment, but the difference between the rose oil and placebo groups was not statistically significant (P=0.99). IBDQ-9 score also increased in both interventions before and after the treatment (P=0.012), though the differences between these two groups were not statistically significant (P=0.61). There were no significant differences between the two study groups either in terms of calprotectin level (P=0.219).

**Conclusion:**

This study showed that rose oil might improve ulcerative colitis clinical outcomes, but for a better evaluation, it is imperative to conduct experiments with a large sample size and longer follow-up observation.

## Introduction

Ulcerative colitis (UC) is a chronic, relapsing-remitting inflammatory disease of the intestine. It is the most common form of inflammatory bowel disease (IBD) worldwide characterized by inflammatory changes and superficial ulcerations in the rectum and colon [1, 2]. UC has distinct pathological and clinical features, but its etiology and exact pathogenesis are unknown. Symptoms of this disease can include increased defecation frequency, rectal bleeding, urgency or tenesmus, abdominal cramps and pain, depending on the extent and severity of the disease. UC can seriously impact the health-related quality of life (HRQoL) of patients and has significant social, psychological, and financial repercussions [3, 4]. UC treatment aims to induce and maintain clinical remission, improve HRQoL, and mucosal healing [1, 5]. The first-line therapy involves 5-aminosalicylate (5-ASA) compounds – which have been successful in approximately 50% of patients — and next line therapy includes steroids, immunosuppressants, and biological drugs that have risks of infection and malignancy [2, 4, 6]. Therefore, it is imperative to explore new and safe remedies with a natural origin as a complementary/alternative therapy for this disease [7].

Rose oil is one of these remedies, which is prepared by soaking the whole petals of damask rose (*Rosa damascena*) in sesame oil as the carrier. In traditional manuscripts, rose oil with this method of preparation has been considered as an anti-inflammatory agent especially in the gastrointestinal tract [8, 9]. The *R. damascena* Mill L. –commonly known as damask rose—is known as *Gol-e Mohammadi* in Iran. It is one of the most important species of the Rosaceae family [10]. *R. damascena* has traditionally been used as a hypnotic, cough suppressant, anti-inflammatory, anti-ulcer, gentle laxative agent, and for treating digestive problems [11, 12]. At present, in addition to its perfuming effects, flowers, petals and hips (seed-pot) of this plant are also used for medical purposes. New studies have proven the effectiveness of the components of damask rose and rose oil in animal models of IBD and inflammation [13, 14]. UC is a recurrent and refractory disease that has no permanent cure, so it can result in significant long-term morbidity, physical and psychological discomfort, thereby affecting the quality of life (QoL) of patients [1]. Furthermore, its treatment is expensive (especially biological drugs), and medications may have serious side effects [15]. Accordingly, the discovery of novel complementary/alternative drugs including those from natural sources that overcome the abovementioned drawbacks of the current therapy is of great interest [16]. The aim of this study was to evaluate the efficacy of rose oil in comparison with placebo in controlling symptoms of UC.

## Materials and Methods

### Patients

The patients were chosen from those who had a clinical diagnosis of UC based on colonoscopy and allied pathology report. They were visited by a gastroenterologist with more than 15 years of experience at the beginning of the trial.

### 2. Sample Size Calculation

Considering alpha=0.05, the power of study equal to 80%, and mean of partial Mayo clinic score of 4 with a standard deviation of 2, we hypothesized that our intervention would show a 25% accepted effect size. Thus, the sample size was determined as 20 patients in each group with 1:1 allocation ratio.

### Inclusion and Exclusion Criteria

Patients with definite diagnosis of UC who showed moderate to severe disease based on the diagnostic criteria (classified using the partial Mayo clinic score as reference) and previously confirmed by colonoscopy were recruited in the trial. All patients were above 18 years old, and there were no limitations on gender. All patients volunteered to sign an informed consent. Exclusion criteria included as follow; 1. Patients who refused to provide a signed informed consent for participating in the study; 2. Patients with a history of using steroids for other diseases within four weeks before entering the current study; 3. Patients with a history of consuming any non-steroidal anti-inflammatory drugs within one week before entering the study; 4. Patients with a history of antibiotic consumption or any medicinal plants within two weeks before entering the study; 5. Patients with a positive history of allergy or hypersensitivity to any component of the rose oil or herbal medicine; 6. Patients who were in the lactation period or pregnant.

The patients whose symptoms worsened, those with serious adverse reactions during treatment, or patients who voluntarily quit or were found to be ineligible for the study by the investigators were also excluded from the study. If the patient did not pass the treatment period, they would be excluded from the study. Other exclusion cases were due to new positive pregnancy test and personal request due to health considerations.

### Randomization, Blinding, and Allocation Concealment

A package of medication had been considered for each patient with a numeric label containing soft capsules for consumption for two months. The medications for both groups were produced by Barij Essence Pharmaceutical Co. (Iran) and also randomly allocated based on a randomized list. They were completely the same in terms of color, shape, and packaging. Concealment was done by a research assistant who was not aware of the study protocol. The randomized list was generated using Microsoft Excel 2010, USA with a block randomization method. Accordingly, 40 eligible participants were also assigned randomly into two parallel groups with a 1:1 allocation ratio. The participants, gastroenterologist, medical researcher assistant, and statistician were blinded to the allocation of the patients.

### Treatment Protocol

Treatments consisted of a soft capsule 1000 mg to be taken orally. One group received rose oil soft capsule, and the other received placebo soft capsule filled with liquid paraffin. Both soft capsules were the product of Barij Essence Pharmaceutical Company. Patients in both groups were given two capsules three times daily half an hour before meal. The treatment duration for both groups was two months. During the study, the patients were allowed to take concomitant or current medications for UC. The patients were followed for two months and visited three times, once on arrival and twice at the end of each month. The questionnaires about patients’ demographic data and disease course were filled out at the baseline visit. Clinical symptoms and QoL of the patients were evaluated by partial Mayo clinic standard form and Persian version of IBD questionnaire-9 (IBDQ-9) [18]. The partial Mayo clinic standard form was filled at the first visit and then at the end of each month by the medical research assistant. Also, the IBDQ-9 form was completed at the beginning of the study and after two months of treatment by the medical research assistant. By the final visit, the questionnaires about patient’s satisfaction and ease of usage were filled out. Any adverse events, recorded in a diary, were collected by a medical research assistant at the last appointment. All the history takings were done by the medical research assistant, and all the participants were examined by one expert gastroenterologist.

### Primary Outcomes

The primary outcome measures included: 1. Clinical symptoms before and after treatment, as evaluated by partial mayo clinic score [17]; 2. QoL scores before and after the treatment, as evaluated by IBDQ-9 with proven validity and reliability in previous studies; IBDQ-9 is a specific QoL questionnaire in IBD patients, which is under usage in more than 10 languages. It contains the following domains: bowel symptoms, emotional status, systemic symptoms, and social function [18]. 3. Calprotectin levels before and after treatment assessed by fecal calprotectin using a commercially available enzyme-linked immunosorbent assay (Buhlmann Laboratories AG, Schonenbuch, Switzerland) [19].

### Data Collection

This study was a single-center, 2-arm, randomized, double-blind, placebo-controlled clinical trial conducted in Motahari Clinic of Shiraz University of Medical Sciences, gastroenterology clinic, the main academic center of southern Iran, from October 2016 to November 2017. The data collection was conducted in this period according to the trial design.

### Secondary Outcomes

The secondary outcome measures involved evaluating the patients’ satisfaction and the number of participants with any adverse events. The patients’ satisfaction was assessed by a visual analog scale, ranging from 1 (least satisfactory) to 10 (most satisfactory). The adverse events were recorded on a diary during the treatment time.

### Ethical Issues

The current study was registered at the Iranian Registry of Clinical Trials (IRCT registration number: IRCT2016120323823N2). All participants provided a signed informed consent form before the trial. Furthermore, the Ethics Committee of Shiraz University of Medical Sciences approved the protocol for this study (reference number: IR.SUMS.REC.1394.211), and there were no significant changes during the experiment.

### Statistical Analysis

The data were analyzed via the SPSS software (IBM Corp, Released 2013, IBM SPSS Statistics for Windows, version 22.0 Armonk, NY). The frequency (percent), mean ± standard deviation (SD) and median were used for the data description. Independent sample *t*-test, Mann-Whitney, and Chi-Square test were used for comparing the difference between the two groups. Wilcoxon signed-rank test and paired sample* t*-test were applied to compare the difference within an intervention group. Analysis of covariance (ANCOVA) was also used for comparing both groups adjusted for the baseline variables. The differences were considered statistically significant when the P-value was <0.05.

## Results

**Figure 1:**
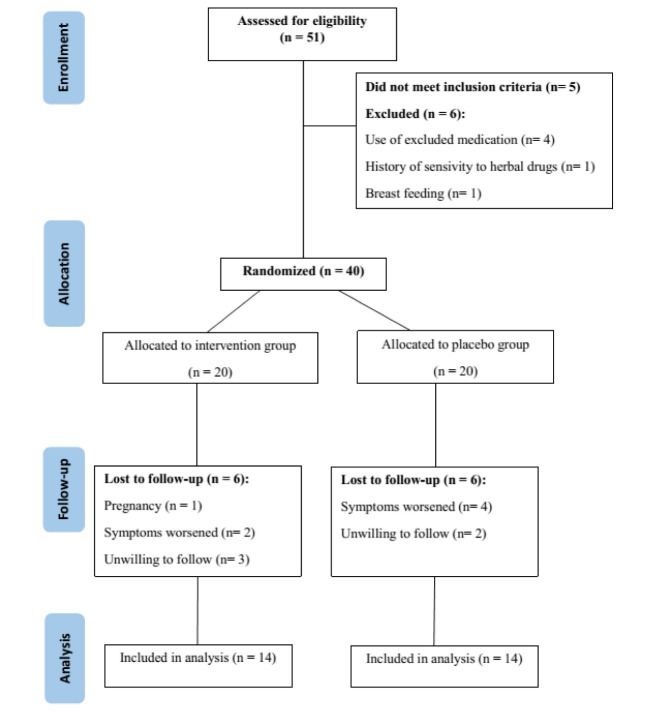
Consort flow diagram of the trial

A total of 40 patients were divided into two groups: 20 patients in the rose group and 20 patients in the placebo group. Finally, 28 patients (14 in the rose oil group and 14 in the placebo group) completed the study. Twelve participants (30%) withdrew from the study; 9 of them did not come for the second visit (5 in the rose group and 4 in the placebo group), and 3 patients did not come for the third visit (Figure-1). All patients completed the baseline screening. There was no statistically significant difference between these two groups in terms of baseline demographic characteristics (age and gender), disease course (duration, number of admissions, extent of the disease), clinical symptoms (defecation frequency and rectal bleeding), partial Mayo clinic score, and IBDQ-9 (P>0.05) at baseline visits. It suggests that the baseline data for the groups were balanced and comparable (Table-1). Concomitant medication was also similar in both study groups without statistically significant difference (P=0.77). Partial Mayo clinic score significantly decreased in both groups after the treatment (P<0.05), but the differences between these two groups were not statistically significant (P>0.05, Table-2). IBDQ-9 revealed a significant improvement after the treatment compared with the baseline levels in both groups (P<0.05), but the differences between these two groups were not statistically significant (P>0.05, Table-2). Mean of fecal calprotectin levels decreased in both groups at the end of the study; however, this difference was not statistically significant ( Table-3). The between-group analysis also showed no significant difference after the trial (P=0.219). Although there was greater patients’ satisfaction in the rose group (7.46 ± 2.33 vs. 6.79 ± 2.01), the statistical analyses showed no significant difference between these groups (P =0.426, Table-4). Among 20 patients treated with rose oil, 2 (10%) withdrew from treatment because of gastrointestinal adverse events, as compared with 3 of 20 patients receiving liquid paraffin (15%). The statistical analysis indicated no significant difference between the two study groups in terms of side effects (P=0.56). Increases in defecation frequency (3 patients) and abdominal cramp (2 patients) were the most common side effects that the participants noted.

## Discussion

Rose oil soft capsules significantly decreased partial mayo clinic and IBDQ-9 scores at the end of the study, though this change was also observed in the placebo group. The fall detected in calprotectin levels –an objective sign of ameliorating inflammation mediators— was not statistically significant. Flowers and petals of damask rose contain flavonoids, polyphenols, and vitamin C, and it has been reported that flavonoids and polyphenols have anti-inflammatory effects, especially in the gastrointestinal tract [20]. Anti-inflammatory properties of flavonoids have been demonstrated in previous experimental studies. Flavonoids have also shown anti-ulcer effects [21, 22]. Oral administration of geraniol (one of the components of rose oil) has been a potential therapeutic agent for experimental colitis in mice and significantly reduced colitis clinical signs and pro-inflammatory indices [14]. Another animal study revealed that geraniol with its antioxidant and anti-inflammatory properties improved experimental colitis in rats [13]. Also, the sesame oil– which is used as a carrier for preparing rose oil– is effective in the animal model of colitis and inflammation. Oral administration of sesame oil accelerated the healing of an inflamed colon in rats [23]. Another study indicated that sesamol (one of the active constituents of sesame oil) has mucosal protective effects for IBD in rats [24]. These findings may justify the promising effect of rose oil soft capsules for relieving partial Mayo clinic and IBDQ-9 scores at the end of the study. In spite of large administration of herbal remedies for curing UC and its symptoms, shortage of high-quality design clinical studies proving the efficacy and safety of these products is obvious [25, 26]. In a multicenter placebo-controlled study on *Andrographis paniculata* extract at an oral dose of 1800 mg, a relatively acceptable clinical response was reported by Sandborn* et al.*[27]. The authors prescribed this product for eight weeks to mild to moderate UC patients in five different countries [27]. Our findings are in line with this study, but it should be noticed that this herb is not an endemic plant, which was imported from neighboring countries years ago [28]. Langmead *et al.* [29] revealed the positive effect of oral consumption of aloe vera gel in 44 UC patients. The results were similar to ours in term of clinical outcomes and safety profile considering their shorter treatment period (four weeks), though they explored histological aspects too [29]. The difference between active and placebo groups was not statistically significant in both investigations. Gong* et al.* [30] examined a traditional Chinese medicine formula (Fufangkushen colon-coated capsules) in comparison to mesalazine for eight weeks. The results indicated that this remedy could be an effective and safe option, though the control group revealed the same response [30]. The results are in accordance with ours in the abovementioned aspects, although the sample size was larger in the Chinese group (320 vs. 39 patients). Both rose oil soft capsule and placebo (liquid paraffin) groups showed promising results in this survey. It may be attributed to the effects of preferred placebo. As the safety of the placebo was so imperative for the authors, the item selected for the control group was not very optimal. Paraffin (mineral oil) has shown partially accepted effects for increased bowel movements in children [31]. So, it may affect the inflammatory features of the disease, causing fewer symptoms in UC patients. Even though we preferred a choice of treatment with an inclusive analysis profile (rose oil capsule), we did not recognize the definite active ingredient(s) to explore the mechanism of action in this piece of research –a common weakness in such trials [26, 32]. Consideration of holistic approach in traditional and complementary/alternative medical systems could well explain this shortcoming. The synergistic effect of bioactive substances is also a superior rationale for convincing the pessimistic researchers [33]. Alternatively, finding the main active ingredient was not set as an outcome for our investigation and could be scrutinized in future pharmacological studies. We used calprotectin for an objective examination of our hypothesis that showed a descending trend in the course of our study. However, this was not statistically significant while observing inner and between-group differences. Notably, the power of these data was more than 97%. This shows that such a small sample size is undoubtedly satisfactory for this item. At present, traditional/complementary and alternative modalities have are gaining popularity in different medical fields. Use of these options for the treatment of UC, as adjunctive therapy beside conventional medications, and as a holistic approach in the therapeutic process is very common nowadays [32]. Persian medicine (PM) –sometimes called *Unani medicine*— one of the most acknowledged medical systems from the medieval period, has been under the practice in Iran and many countries for a long history [34]. Based on PM perspective, temperaments and humors should be kept in mind while treating the patients, thus, considering these factors rose oil could act as a tonic agent in large intestine and could throw out the waste material including excess unbalanced humors from the site [11, 35]. On the other hand, rose oil could play a role as a soothing agent, and consequently, it could adequately decrease the symptoms of local inflammation [9]. Although anti-inflammatory and ulcer-healing effects of *R. damascena* have been demonstrated in several studies, and the effects of some components of this plant and the rose oil on experimental colitis have been shown in some animal studies [14, 36], to the best our knowledge, there is no clinical trial study on the efficacy of rose oil in treating UC. Acceptable safety profile can be mentioned as an advantage of the current study, as there were no harmful or life-threatening side effects during the treatment period. Gastrointestinal symptoms were the chief complaint in these groups causing them to voluntary quit the study. The participants were also satisfied with the suggested medicines in both groups, though this item did not show any significant difference. Our study had some limitations; relatively small sample size and short-time follow-up were the main limitations of our study. A glance at the results of study reveals that the powers of these data are low indeed. This means if the sample size of the study grows, it may result in significant differences between the two products. Use of an affordable product which has gone through all legal and scientific procedures in terms of quality control profile from relevant authorities was an important superiority in this study.

## Conclusion

As mentioned previously, both rose oil and placebo may be effective for UC. However, comparing the effects of these two products, no significant difference was observed between the two study groups at the end of the study. This study was a pilot study with a small research population. Experiments with larger sample populations through multicenter clinical investigations with follow-up observation should be conducted to validate the findings of this study.

## Acknowledgment

This work was supported through a collaborative project between Shiraz University of Medical Sciences and Barij Essence Pharmaceutical Company (grant number: BE-113 ). This study was a part of the Ph.D. dissertation of Dr. Ali Tavakoli for the Iranian Board of Traditional Medicine (94-01-64-10329). We would also like to extend our thanks to Dr. Hossein Molavi for his support in statistical analysis.

## Conflict of interest

The authors declare that there is no conflict of interest. Leila Mohammad Taghizadeh is an employee of Barij Essence Pharmaceutical Company.

**Table 1:** The Baseline Demographic and Primary Characteristics in Both Intervention Groups

**Variables**	**Total**	**Group**		**P-value**
		**Rose (n=14)**	**Placebo (n=14)**	
**Age (years)**				**0.499** †
Mean ± SD	41 ± 10	40 ± 10	42 ± 11	
**Gender**
Female	13 (46.4 %)	5 (35.7 %)	8 (57.1 %)	**0.225** *
Male	15 (53.6 %)	9 (64.3 %)	6 (42.9%)	
**Duration of UC**
< 5 years	9 (32.1 %)	6 (42.9 %)	3 (21.4 %)	**0.376** ‡
5 y ≤ and < 10 y	8 (28.6 %)	2 (14.2 %)	6 (42.9 %)	
≥10 years	11 (39.3 %)	6 (42.9 %)	5 (35.7 %)	
**Hospital Admission (n)**
Yes	9 (32.1 %)	4 (28.6 %)	5 (35.7 %)	**0.5** *
No	19 (67.9 %)	10 (71.4 %)	9 (64.3 %)	
**Severity of diagnosis**
Proctitis	5 (17.9 %)	2 (14.3 %)	3 (21.4 %)	**0.689** ‡
Left. sided Colitis	8 (28.6 %)	4 (28.6 %)	4 (28.6 %)	
Extended Colitis	3 (10.7 %)	2 (14.3 %)	1 (7.2 %)	
Pan-Colitis	12 (42.8 %)	6 (42.8 %)	6 (42.8 %)	
**Stool frequency**
Normal	1 (3.6 %)	0 (0.0 %)	1 (7.1 %)	**0.667** ‡
1-2 times more	14 (50.0 %)	7 (50.0 %)	7 (50.0 %)	
3-4 times more	9 (32.1 %)	5 (35.7 %)	4 (28.6 %)	
5 times more	4 (14.3 %)	2 (14.3 %)	2 (14.3 %)	
**Rectal bleeding**
Normal	19 (67.8 %)	9 (64.3 %)	10 (71.5 %)	**0.635** ‡
Bleeding < 1-2	5 (17.9 %)	2 (14.3 %)	3 (21.4 %)	
Bleeding > 1-2	3 (10.7 %)	2 (14.3 %)	1 (7.1 %)	
Always Bleeding	1 (3.6 %)	1 (7.1 %)	0 (0.0 %)	
**Physician global assessment**
Mild	10 (17.9 %)	7 (50.0 %)	3 (21.4 %)	**0.210** ‡
Moderate	13 (28.6 %)	5 (35.7 %)	8 (57.2 %)
Severe	5 (10.7 %)	2 (14.3 %)	3 (21.4 %)
**IBDQ - 9**
Mean ± SD	41.6 ± 9.5	41.6 ± 9.5	44.6 ± 9.4	**0.408** †
**Partial Mayo Clinic Score**
Mean ± SD	3.93 ± 2.24	3.93 ± 2.24	3.86 ± 1.46	**0.921** †

† based on t-test * Based on Chi-Square test ‡ Based on Mann-Whitney test.

**Table 2:** The Differences of Primary Outcome Measures Between 2 Intervention Groups

**Parameters**	**Time**	**Group**		**Diff.**	**95% CI**		**P- value**
		**Rose (n=14)**	**Placebo (n=14)**		**Lower**	**Upper**	
**Stool frequency**
	Baseline	1.64 ± 0.75	1.50 ± 0.86	0.14	-0.48	0.77	0.667 ‡
	After 1M	1.14 ± 0.54	1.0 ± 0.56	0.14	-0.28	0.57	0.603
	P-Within^ᴥ^	0.020	0.035
	After 2M	0.86 ± 0.77	0.93 ± 0.62	-0.07	-0.61	0.47	0.769
	P-Within^ᴥ^	0.021	0.074
**Rectal bleeding**
	Baseline	0.64 ± 1.01	0.36 ± 0.63	0.28	-0.37	0.94	0.635 ‡
	After 1M	0.29 ± 0.61	0.14 ± 0.54	0.14	-0.30	0.59	0.571
	P-Within^ᴥ^	0.102	0.257
	After 2M	0.14 ± 0.36	0.07 ± 0.27	0.07	-0.18	0.32	0.769
	P-Within^ᴥ^	0.066	0.102
**Physician global assessment**
	Baseline	1.64 ± 0.75	2.0 ± 0.68	-0.36	-0.91	0.2	0.210 ‡
	After 1M	1.36 ± 0.63	1.43 ± 0.76	-0.07	-0.61	0.47	0.910
	P-Within^ᴥ^	0.046	0.011
	After 2M	1.14 ± 0.77	1.14 ± 0.86	0.0	-0.64	0.64	1
	P-Within^ᴥ^	0.068	0.014
**IBD-Q9**
	Baseline	41.6 ± 9.5	44.6 ± 9.4	-3.0	-10.3	4.3	0.408 †
	After 2M	47.5 ± 8.3	48.9 ± 6.5	-1.4	-7.2	4.4	0.617
	P-Within^¶^	0.03	0.012
**Partial Mayo Clinic Score**
	Baseline	3.93 ± 2.24	3.86 ± 1.46	0.07	-1.40	1.54	0.921 †
	After 1M	2.79 ± 1.37	2.57 ± 1.34	0.21	-0.84	1.27	0.679
	P-Within^¶^	0.014	0.013
	After 2M	2.14 ± 1.61	2.14 ± 1.46	0.0	-1.19	1.19	1
	P-Within^¶^	0.022	0.014

**CI:** Confidence Interval, **Pre:** at the starting of the study, **after 1M:** 1 month after using drugs and **after 2 M:** 2 months after starting the study (end point of the study). † Based on t-test ‡ Based on Mann-Whitney test ¶ Based on Paired t-test ᴥ Based on Wilcoxon Signed rank test

**Table 3:** The Comparison of the Patients’ Calprotectin Level in Intervention Groups

**Groups**	**Before intervention** **(Mean ± SD)**	**After intervention** **(Mean ± SD)**	**P-value†**
**Rose oil group**	64.21 ± 93.47	34.75 ± 89.45	0.229
**Controlled group**	67.56 ± 138.19	33.45 ± 2.72	0.122

† based on paired t-test

**Table 4:** The Comparison of the Patients’ Satisfaction between Intervention Groups After Two Months

**Parameter**	**Time**	**Groups**		**Diff**	**95% CI**		**P-value†**
		**Rose oil (n=14)**	**Placebo (n=14)**		**Lower**	**Upper**	
**VAS**	At the end of study	7.46 ± 2.33	6.79 ± 2.01	0.67	-1.05	2.39	0.426

CI: Confidence Interval † Based on t-test
